# Classification of Clear Cell Renal Cell Carcinoma based on Tumor Suppressor Genomic Profiling

**DOI:** 10.7150/jca.50462

**Published:** 2021-02-22

**Authors:** Weimin Zhong, Fengling Zhang, Chaoqun Huang, Yao Lin, Jiyi Huang

**Affiliations:** 1 The Fifth Hospital of Xiamen, Xiamen 361101, Fujian Province, China.; 2 Key Laboratory of Optoelectronic Science and Technology for Medicine of Ministry of Education, College of Life Sciences, Fujian Normal University, Fuzhou 350117, Fujian Province, China.

**Keywords:** clear cell renal cell carcinoma, tumor suppressor genes, molecular subtype, tumor microenvironment, compounds, drug sensitivity, immunotherapy

## Abstract

Clear cell renal cell carcinoma (ccRCC) is the most prevalent type of malignancy in adults. However, the clinical significance of tumor suppressor genes (TSG) is largely elusive. Herein, the expression profile TSGs and its clinical response in ccRCC were investigated. A total of 603 ccRCC samples from two cohorts (TCGA and ICGC) were retrieved in this study. Three molecular subtypes (C1, C2, and C3) were identified based on the TSGs expression profile in the TCGA dataset. Through Weighted Gene Correlation Network Analysis (WGCNA), six modules associated with three subtypes were identified. Pathway enrichment for the modules revealed that crucial pathways including p53 signaling and immune-related pathways were significantly enriched. We further focused on the relationship between immune infiltration level and subtypes, and found that subtype C1 was associated with higher immune infiltration level, subtype C2 was corresponding with medium immune infiltration level, whereas subtype C3 was correlated with lower immune infiltration level. Interestingly, C2 have a better survival outcome, while C1 and C3 showed a poor prognosis. Considering their survival difference, we then performed a differentially expression analysis between C2 and C1&3, and a total of 99 differentially expressed tumor suppressor genes (DETSGs) were identified. According to these DETSGs, 59 potential compounds with 28 mechanisms of action (MOA) were predicted using the Connectivity Map (CMap) database. Among these compounds, leflunomide, naftopidil, and ribavirin were the most prospective compounds for the treatment of ccRCC. In addition, we found that subtype C2 is more sensitive to sorafenib and sunitinib drugs, and C2 have more likelihood to be responded to immunotherapy. In summary, the three subtypes hinged on the tumor suppressor gene expression for ccRCC might contribute to understanding the underlying molecular mechanisms of ccRCC. Also, its potential compounds might offer guidelines for developing a novel treatment strategy of ccRCC.

## Introduction

Renal cell carcinoma (RCC) is listed among the 10 most common types of tumors in adults, responsible for 2%-3% of adult malignancies 1. Clear cell renal cell carcinoma (ccRCC) forms the vast majority of RCC accounting for 70% of RCC cases. The remaining include papillary (10-15%), and chromophobe (5%) carcinomas 2, 3. Using abdominal imaging, patients with ccRCC can be detected 4. However, the treatment for ccRCC through radiation and chemotherapy is insensitive and the survival rate of 5-years in advanced stage is < 10%, particularly in metastatic ccRCC cases 5. In metastatic ccRCC, immunotherapy with interleukin-2 (IL-2) and interferon (IFN) are conventional treatments despite exhibiting many side effects and limited efficacy 6. In 2005 and 2006, sorafenib and sunitinib were approved as targeted therapy for metastatic ccRCC, and so far, they have achieved some progress 7, 8. However, they are limited for target therapies and prone to drug resistance 9.

Recently, with the advent of next-generation sequencing and microarray technologies, several tumor suppressor genes (TSGs) were identified via the bioinformatics analysis and series of experiments. Tumor suppressor genes maintain genome integrity, regulates cell proliferation, differentiation, and apoptosis 10. Numerous studies reported that a combination of TSGs and conventional therapies achieved significant progress, particularly the *p53* gene, which yielded synergistic therapeutic benefits 11, 12. Nonetheless, given the large number of TSGs, the subject on relationship between TSGs and cancers is largely understudied.

In this work, we aimed to comprehensively investigate the association between TSG phenotype and ccRCC. The consensus clustering algorithm was applied to categorized 512 patients into three molecular subtypes and further validated in the ICGC cohort. Through WGCNA analysis, six modules were identified then their potential roles were evaluated via GO and KEGG enrichment analysis. Additionally, the relationship between tumor microenvironment and subtypes was investigated, and we found that subtype C1 corresponded to high immune infiltration level, subtype C2 was associated with mid immune infiltration level, whereas subtype C3 related to lower immune infiltration level. Moreover, potential compounds between subtype C2 and subtype C1&3 were predicted using the CMap database, and three compounds including ribavirin, leflunomide, and naftopidil were considered as promising drugs to the treatment of ccRCC. These findings might help in the identification of novel markers to subdivide ccRCC patients more precisely and enhance the treatment of ccRCC patients.

## Materials & Methods

### Extraction of tumor suppressor genes and ccRCC samples

1217 TSGs were downloaded from the TSGene database (https://bioinfo.uth.edu/TSGene/) 13. The RNA data, clinical data, and TSGs in ccRCC were retrospectively collected from the TCGA database (https://cancergenome.nih.gov/) 14. The expression of TSGs with 512 ccRCC samples were screened via the following criteria: (a) patient with complete clinical information, (b) patients with survival time need > 30 days, and (c) genes with expression levels >0 in each ccRCC sample. In addition, a total of 91 samples with expression data of TSGs in the validation dataset were collected.

### Identification of ccRCC subtypes based on the expression of tumor suppressor genes

The “ConsensusClusterPlus” package was used to identify the potential molecular subtypes of ccRCC based on the expression of TSGs in the TCGA dataset and ICGC dataset, respectively 15. The similarity distance between samples was calculated according to the Euclidean distance and the k-means method was used for clustering 16. The clustering was conducted using 1000 iterations, each iteration containing 80% samples, and the optimal cluster number was selected based on cumulative distribution function (CDF) curves of the consensus score 17. The top 100 variance of TSGs were selected to perform PCA analysis in the TCGA dataset and ICGC dataset respectively. In addition, the probability of clinical response to immunotherapy and immune checkpoint blockade was predicted by the Tumor Immune Dysfunction and Exclusion (TIDE, http://tide.dfci.harvard.edu/) analysis and subclass mapping (SubMap, https://cloud.genepattern.org/gp/pages/index.jsf) analysis. To further explore the genomic alteration among the three subtype, we applied to the “maftools” R package to investigate the SNP density of the subtypes (https://www.bioconductor.org/packages/release/bioc/html/maftools.html). The molecular function of the subtype was further identified using the “GSVA” R package (https://bioconductor.org/packages/release/bioc/html/GSVA.html).

### Gene co-expression network analysis

Using the “WGCNA” R package, the common pathways associated with gene modules were identified 18. The network construction and module determination were performed using an unsigned type of topological overlap matrix (TOM) where the soft power of β value was 3, the selected minimal module size was 30, and the branch merge cutoff height was 0.25. GO annotation and KEGG enrichment were conducted through R package “clusterProfiler” and the significant pathway or function was selected with the criterion: adjusted p value <0.05 19.

### Exploring the association between subtype and immune infiltration level

In our previous work, we uncovered that the pathways in the gene module were enriched in immune-related crosstalk. And here, we first quantified the enrichment score of 29 immune signature in each patient by the single-sample gene-set enrichment analysis (ssGSEA) algorithm. Then, ESTIMATE algorithm was adopted to approximate the immune cell infiltration levels (immune score), tumor purity, and stromal content (stromal score) of ccRCC sample (https://bioinformatics.mdanderson.org/estimate/rpackage.html). The Immune infiltration level (immune score, tumor purity, and stromal score) of distinct subtypes was compared through the Kruskal-Wallis test. In addition, we also investigate the expression level of the immune check point in the three subtypes.

### Identification of candidate small molecules

The Connectivity Map (CMap) database (http://www.broadinstitute.org) was used to predict the potential compounds that might reverse or induce biological states encoded in specific gene expression markers 20. To explore the potential activity of small molecules from CMAP database in different subtypes, the up-regulated and down-regulated differentially expressed TSGs (DETSGs) were screened between subtype C2 and subtype C1&3. The up-regulated DEGs and down-regulated DEGs were uploaded to the CMAP database for gene set enrichment respectively. Finally, small molecules with enriched values were ranked from -1 to 1 by calculation. The positive connectivity value (close to +1) suggesting that corresponding small molecules induce subtype gene expression, while negative connectivity value (close to -1) implied that corresponding small molecules could reverse subtype gene expression.

### Prediction of chemotherapeutic response

Genomics of Drug Sensitivity in Cancer (GDSC, https://www.cancerrxgene.org/) database was used to predict the chemotherapeutic response for each ccRCC samples. The two drugs including Sorafenib and Sunitinib that have been approved for the treatment of RCC cases were selected. Through ridge regression analysis in pRRophetic R package, the half-maximal inhibitory concentration (IC_50_) was calculated and the prediction accuracy was determined by 10-fold cross-validation according to the GDSC training set.

### Statistical analysis

The relationship between clinical traits and subtype was analyzed using chi-square test or Fisher's exact test. Multiple testing was corrected by Benjamini-Hochberg's FDR. Kaplan-Meier curves and log-rank tests were used to evaluate the overall survival (OS) rate of the three hub genes. Wilcox test was used to compare the expression value of the hub genes between normal and tumor tissue.

## Results

### Identification and validation of ccRCC subtypes based on the tumor suppressor genes

**Figure 1:**
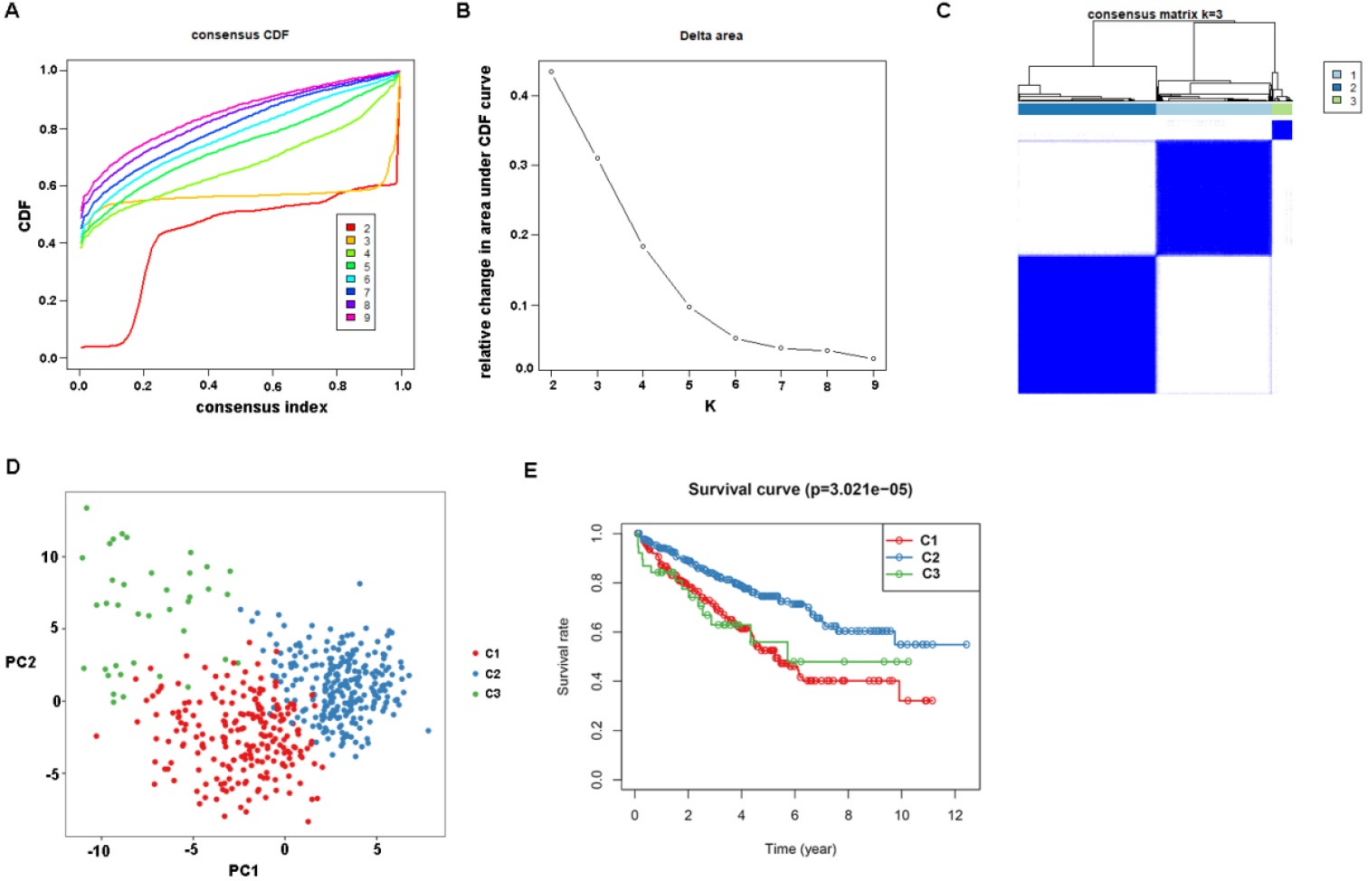
Identification of tumor suppressor gene-related subtypes of ccRCC in TCGA dataset. (A) The cumulative distribution function (CDF) curves, which can described the probability distribution of a real random variable, and established using consensus clustering approach. CDF curves of consensus scores was calculated according to the different subtype number (k = 2, 3, 4, 5, 6, 7, 8, 9). (B) The CDF Delta area curve of ccRCC samples when k=3. (C) The heatmap of consensus matrix for three subtypes obtained by estimating of CDF curves. (D) Principal component analysis (PCA) of gene expression profile of the top 100 variance genes. Each sample is represented with a single point, with different color for each of the three subtypes. (E) Survival analysis for the three subtypes.

**Figure 2:**
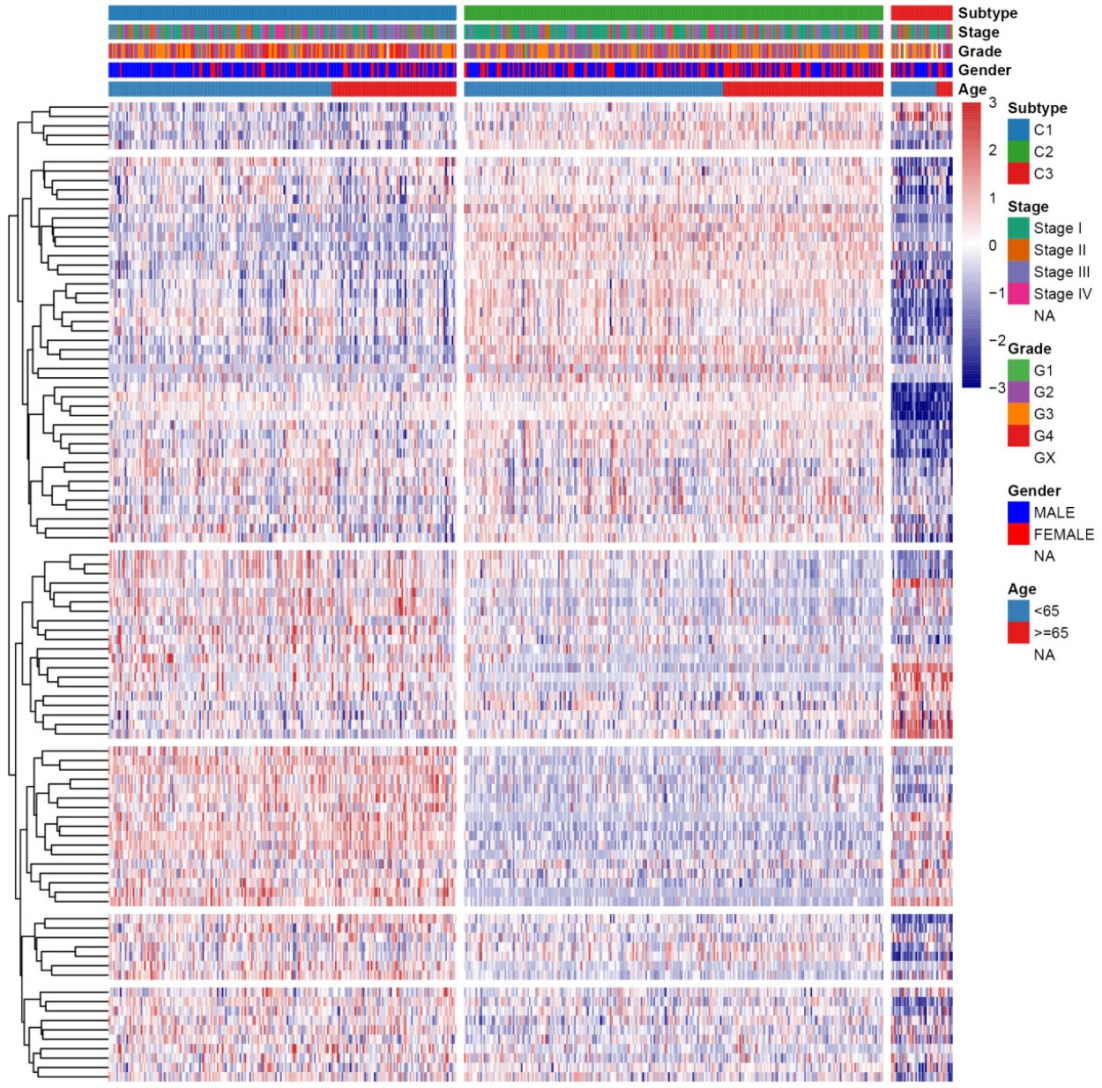
Heatmap and clinical features of the three subtypes in TCGA dataset.

First, the gene expression profile of TSGs was used to explore the potential ccRCC subtypes from the TCGA cohort. All the ccRCC samples were separated into k (k = 2, 3, 4, 5, 6, 7, 8, 9) clusters using “ConsensusClusterPlus” R package. The optimal k value was selected 3 based on the Cumulative Distribution Function (CDF) curves of the consensus score (Figure 1A). Besides, sigClust analysis showed that the consensus cluster (k =3) was significant in all pairwise comparisons (Figure 1B). Therefore, the 512 ccRCC samples extracted from the TCGA cohort were further subdivided into three subtypes underlying the TSGs expression profile (Figure 1C). The top 100 variance of TSGs were further selected for two-dimensional PCA analysis and top two principal components (PC1 and PC2) were extracted and visualized on a scatter plot. As showed in Figure 1D, there are a striking differences in the three clusters. In addition, the results of Kaplan-Meier curve showed that the three subtypes have a significant survival divergence (p < 0.01) (Figure 1E). To further evaluate and validate the stability of the subtypes, an external validation dataset with 91 ccRCC samples was retrieved from the International Cancer Genome Consortium (ICGC) database. By performing the consensus clustering analysis, we further selected k = 3 as the optimal cluster number on the basis of sigClust result and CDF curves (Figure S1). The PCA result suggested that subtype designations were largely consistent with two-dimensional PCA distribution patterns. However, due to the small sample size of the validation dataset, the overall survival rate in the three subtypes was not significant (p > 0.05). To further investigate the clinical significance of clinical traits i.e., age, gender, stage, race, radiation, pharmaceutical, grade, smoking, tumor (T), node (N), metastasis (M), and staging in the three subtypes, chi-SquareTest or Fisher test was performed for each clinical characteristic in the three subtypes. As shown in Table 1, the clinical traits i.e., race, stage, grade, gender, node, and tumor staging showed a significant divergence in subtypes ( p < 0.05), whereas age, radiation, pharmaceutical, smoking have no distinct difference (p >0.05). Moreover, we also discovered that subtype C1 mainly enriched in grade 3 and grade 4 patients, as well as stage III and stage IV patients, comparing with subtype C2 and C3, while the expression level of TSGs were relatively low in subtype C3 (Figure 2). Similar result was also identified in the validation dataset (Figure S2). In addition, we also observed that the subtype C2 account for the most SNP alteration, and C1 ranked second, while C3 have the relative few SNP alteration (Figure S3). Considering subtype C2 have a better survival outcome compare to subtype C1 and C3, we further performed a GSVA analysis between subtype C2 and subtype C1&3 to investigate the potential function of the subtype. We discovered that subtype C2 was associated with the metabolism-related pathway, while subtype C1&3 was related to p53 signaling pathway, ECM receptor interaction (Figure S4).

### Gene co-expression network construction and module annotation

**Figure 3:**
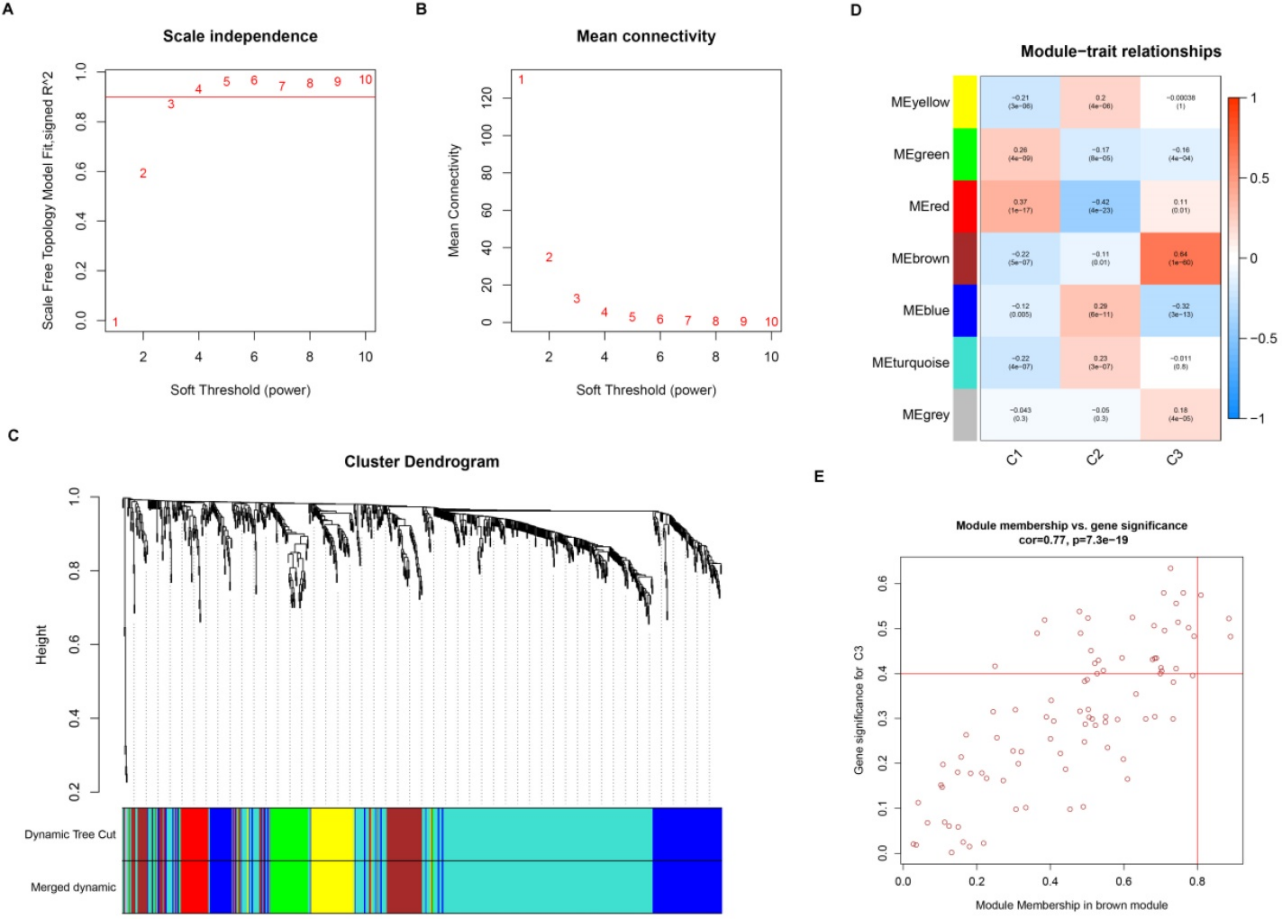
Weighted gene co-expression network of tumor suppressor genes in ccRCC. Analysis of network topology for various soft threshold powers (A, B). (C) Identification of a co-expression module in ccRCC. The branches of the cluster dendrogram correspond to the 7 different modules. Each piece of the leaves on the cluster dendrogram corresponds to a gene. (D) Correlation between the gene module and molecular subtypes. The correlation coefficient was calculated and displayed in each cell between module and subtypes. The corresponding P-value is also annotated. (E) Scatter plot of module eigengenes in the brown module.

Gene co-expression network was constructed to identify biologically significant gene modules and understand the association between genes and ccRCC subtypes. After eliminating outlier samples and excluding low expression level TSGs, a total of 847 TSGs were subjected to cluster analysis and placed in a module (Figure S5). The value of β was selected to 3 (scale-free *R^2^* = 0.90) as the soft threshold to construct a scale-free network (Figure 3A-B). The expression matrix was further converted into adjacency matrix and topological matrix (TOM). According to the TOM, the genes were clustered using the average linkage hierarchical clustering method in accordance with the hybrid dynamic cut tree, the minimum gene size of module was selected 30. The eigengenes were calculated for each module using a dynamic shear method and the closer modules were merged into a new module by setting height =0.25, deepSplit = 2, and minModuleSize = 30. As a result, 7 modules with TSGs were identified using WGCNA methods (Figure 3C). Specifically, the gray module containing genes were not assigned to other modules. Then, the correlation coefficient between modules and three subtypes was calculated. As showed in the Figure 3D, the brown module was most positively correlated with subtype C3 (cor =0.64, *p* = 1e-60), while the red module was most negatively correlated with C2 (cor = -0.42, *p* =4e-23).

**Figure 4:**
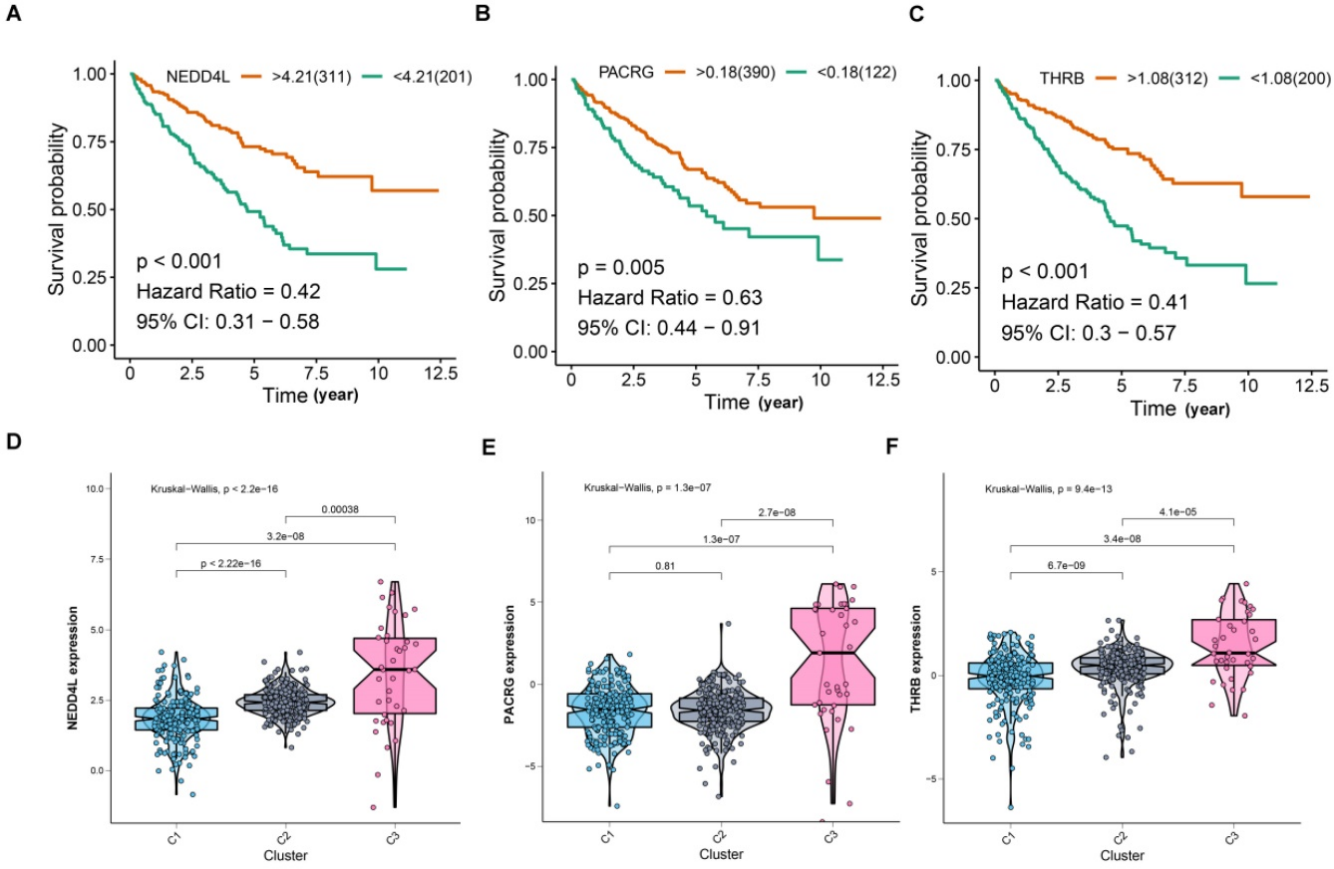
Comprehensive analyses of the three hub genes. (A-C) Kaplan-Meier curves show that the higher expression of hub genes induced better survival outcomes. (D-F) Hub gene expression was compared between three subtypes.

For screening key genes associated with subtype, a threshold was set at: cor. MM > 0.8 and cor. GS > 0.4. Finally, we screened three genes (NEDD4L, PACRG, and THRB) from the modules (Figure 3E). Further survival analysis result revealed that the three protective genes have a significant survival divergence after being grouped based on their expression levels (p < 0.01) (Figure 4A-C). Moreover, the expression level of the three key genes showed a significant difference in the three subtypes. As showed in Figure 4D-F, the expression level of the three genes presented an upward trend in three subtypes, whereby the expression level was highest in the C3, and lowest in the C1.

**Figure 5:**
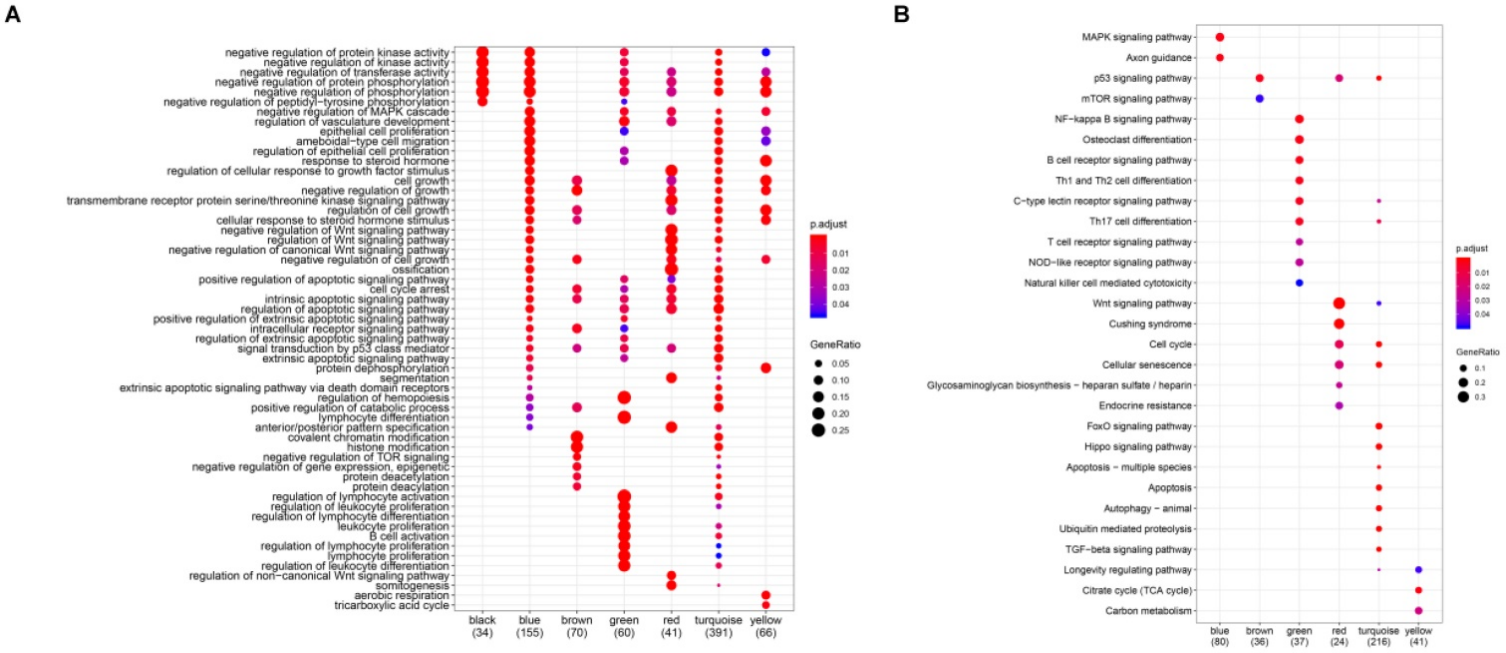
Function enrichment analyses for the gene modules. (A) GO annotation enrichment analyses for the gene modules. (B) KEGG pathway enrichment analysis for the gene modules.

To investigate the biological functions of the six modules (blue, brown, green, red, turquoise, and green), KEGG and GO enrichment analysis were performed. Results from GO analysis revealed that the blue module was mainly enriched in DNA-binding transcription activator activity, RNA polymerase II-specific, cell adhesion molecular binding, the brown module was characterized by the cell adhesion molecular binding, cadherin binding function, the green module was primarily enriched in DNA-binding transcription activator activity, RNA polymerase II-specific, Lys63-specific deubiquitinase activity, the red module was mostly enriched in Wnt-protein binding and insulin-like growth factor binding, the turquoise module was mainly enriched in p53 binding, histone binding, ubiquitin-protein ligase activity and ubiquitin-like protein ligase activity, while the transcription coactivator activity, histone binding and p53 binding were mainly enriched in the yellow module (Figure 5A). Again, results from KEGG analysis showed that the blue module was enriched in MAPK signaling pathway, Axon guidance, transcriptional misregulation in cancer and TGF-beta signaling pathway, the brown module mainly enriched in transcriptional misregulation in cancer and citrate cycle (TCA cycle), the green module mainly enriched in transcriptional misregulation in cancer, NF-kappa B signaling pathway, B cell receptor signaling pathway, PD-L1 expression and PD-1 checkpoint pathway in cancer, Th1, Th2 cell differentiation, Th17 cell differentiation, and C-type lectin receptor signaling pathway, the red module was mainly enriched in wnt signaling pathway, cell cycle, and p53 signaling pathway, the turquoise module was majorly enriched in the cell cycle, p53 signaling pathway, hippo signaling pathway, FoxO signaling pathway, apoptosis, and ubiquitin-mediated proteolysis pathway, and the yellow module mainly enriched in p53 signaling pathway (Figure 5B).

Subsequently, the relationship network of the enriched pathways in these modules was further visualized. As shown in Figure S6, 92 pathways were enriched in these modules. The red and turquoise module has the most common pathways and few interactions of the pathways were enriched in other modules, implying that the red and turquoise modules might have similar functions and molecular mechanism.

### Exploring the relationship between subtype and immune infiltration

**Figure 6:**
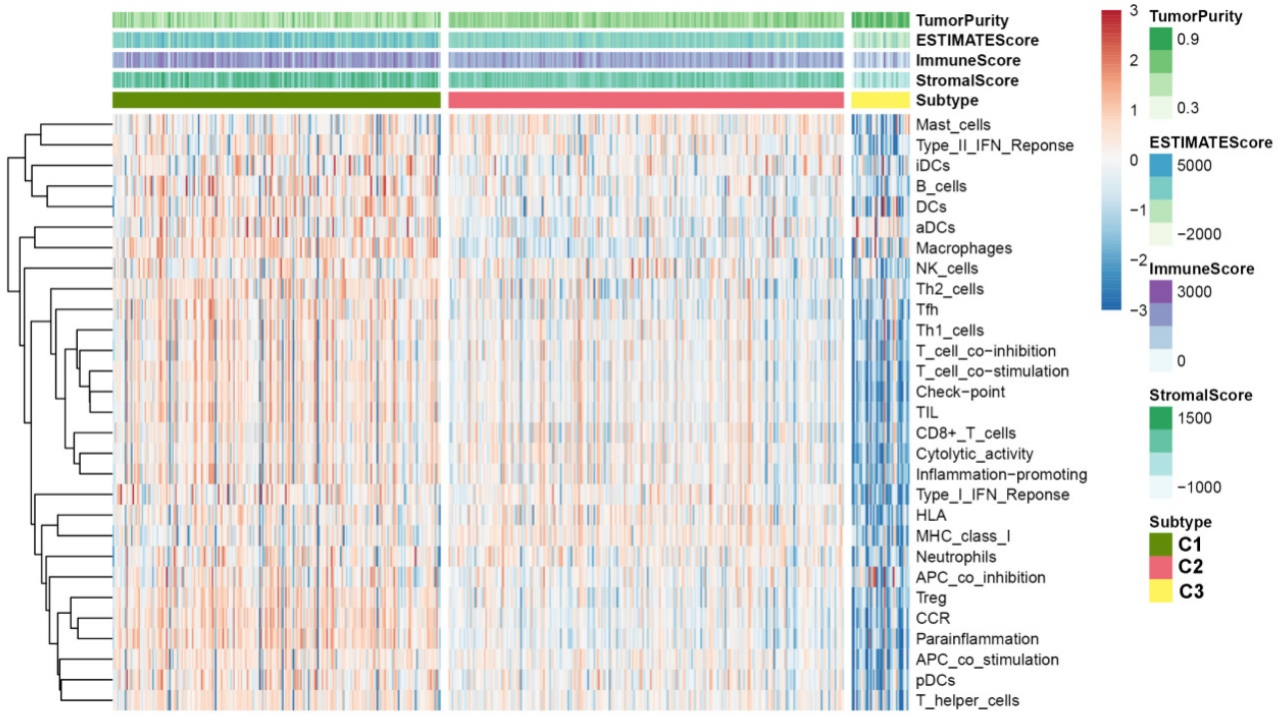
Immune profiles of the three ccRCC subtypes in the TCGA dataset.

**Figure 7:**
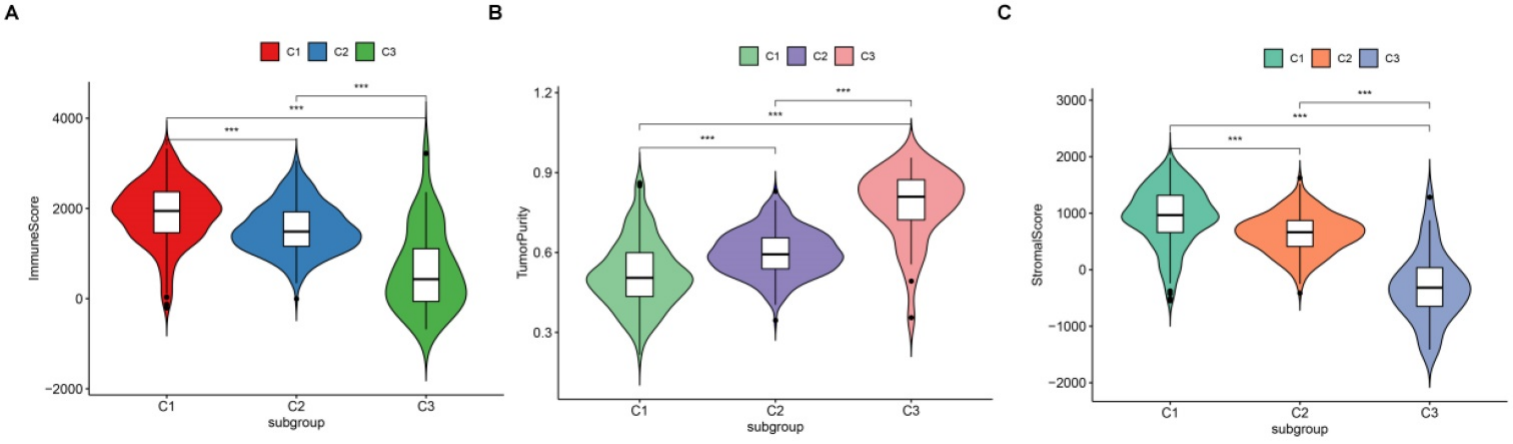
Vioplot for the comparisons of three molecular subtypes in immune score (A), tumor purity (B) and stromal score (C).

Since we have demonstrated that the TSGs in the module involved in the immune crosstalk and cancer-related pathways, a comparison between the immune infiltration in the three subtypes was further conducted. First, 29 immune-associated gene sets including diverse immune cell types, functions, and pathways were analyzed. Then, the ssGSEA score was utilized to quantify the activity or enrichment levels of immune cells, functions, or pathways in the ccRCC samples, and assigned the ssGSEA score to the three subtypes. Interestingly, the three subtypes separated the immune infiltration level, whereby subtype C1 corresponded to high immune infiltration level, subtype C2 corresponded to moderate immune infiltration level, while subtype C3 corresponded to low immune infiltration level (Figure 6). Thereafter, the ESTIMATE algorithm was used to calculate the immune score, stromal score, and tumor purity for each ccRCC sample. As shown in Figure 7A and B, the immune score and stromal score was significantly higher in the subtype C1, while showed a significantly lower level in the subtype C3 (Kruskal-Wallis test, p< 0.01). In addition, a comparison of the tumor purity in the three subtypes presented an opposite trends i.e, the tumor purity was decreasing from subtype C1 to subtype C3 (Kruskal-Wallis test, p< 0.001) (Figure 7C). In summary, these results imply that subtype C1 holds the highest number of immune cells and stromal cells, whereas subtype C3 carries the highest tumor cells. Moreover, we also compared the expression level of several known immune check point among the three subtypes. Interestingly, the overall expression level of immune checkpoint were highly expressed in the C1, while showed a relatively low level in C2 and C3 (Figure S7).

### Connectivity map analysis identifies potential compounds/inhibitors capable of molecular subtypes

**Figure 8:**
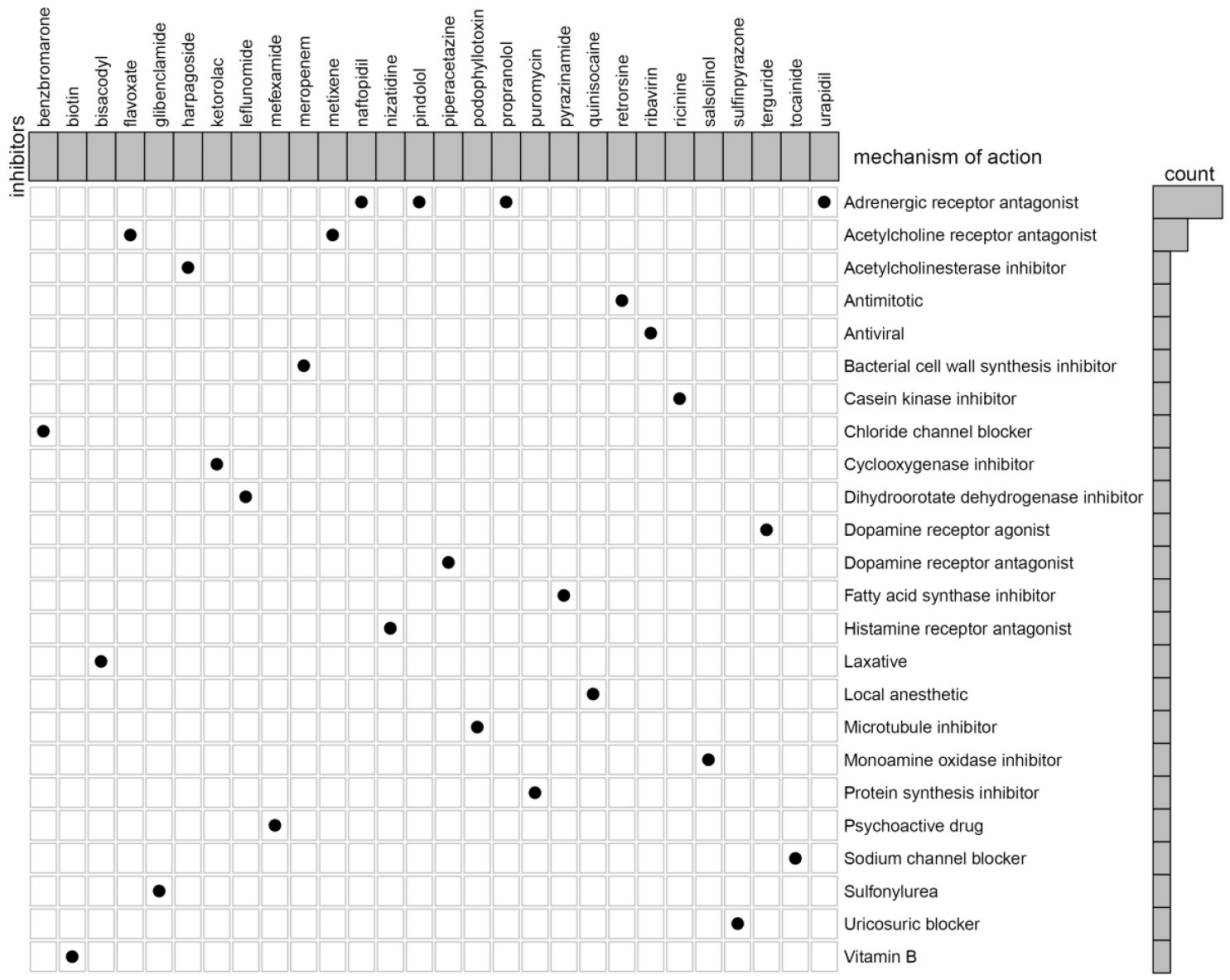
The heat map shows each compound of the shared action mechanism (row) in CMap, sorted in descending order of the shared action mechanism.

In our previous studies, we reported that C3 and C1 were corresponded to poor survival outcomes, while C2 corresponded to better survival outcomes. Therefore, we try to identify the potential compounds or inhibitors for the ccRCC subtypes patients (C2 VS C1&3). First, differential expression analysis between C2 group and other groups (C1&3) was performed using the “limma” R package. The results including 55 up-regulated and 44 down-regulated differentially expressed TSGs (DETSGs) with the cutoff |logFC|>0.58 and FDR < 0.05, respectively (Figure S8). Then, the up-regulated genes and down-regulated genes were uploaded to the CMAP database. As a result, 59 candidate compounds were screened with an absolutely enrichment score of ≥ 0.50 and a *p*-value < 0.05 (Table S1). Among them, leflunomide, naftopidil, and ribavirin were predicted as promising compounds for the treatment of ccRCC. Further, through CMap mode-of-action (MoA) analysis, a total of 28 compounds with 24 mechanisms of action were enriched (Figure 8).

### Immuno/chemotherapies for three molecular subtypes

**Figure 9:**
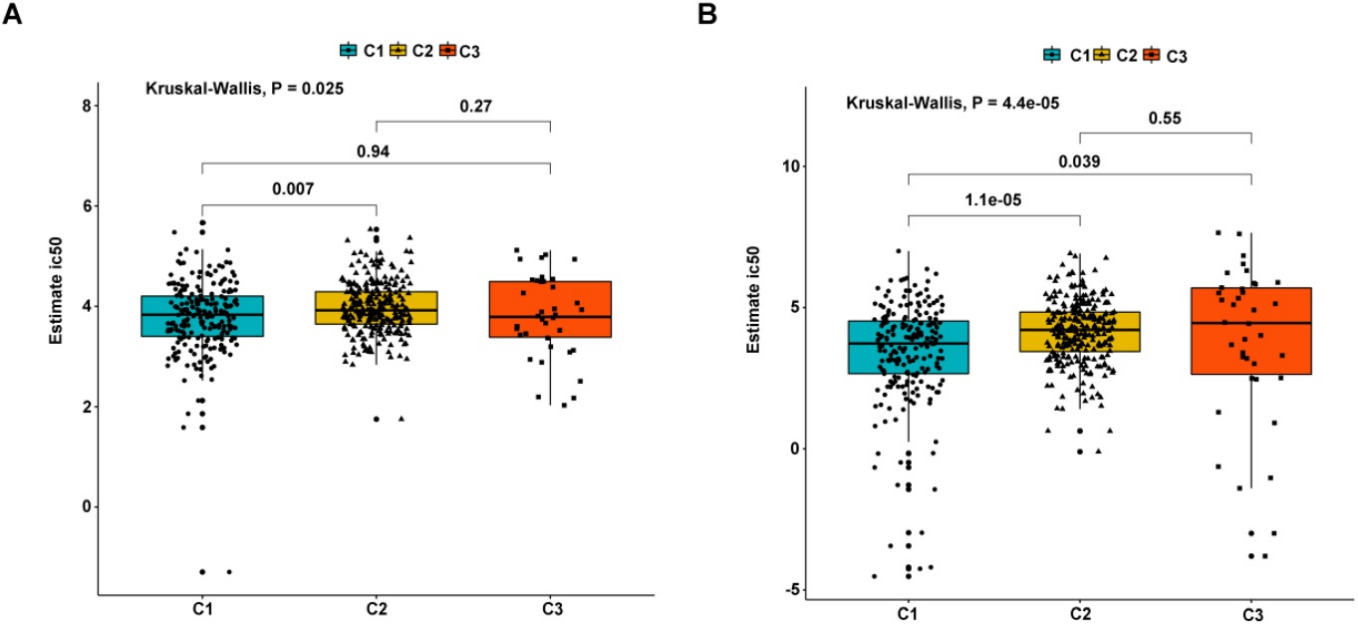
Differential putative chemotherapeutic response to subtypes. (A) The box plots of the estimated IC50 for Sorafenib in subtypes. (B) The box plots of the estimated IC50 for Sunitinib in subtypes.

As earlier mentioned, the sorafenib and sunitinib drugs were approved to be used in the treatment of RCC in 2005 and 2006, respectively. Here, we estimate the sensitivity of the two chemo drugs in three molecular subtypes. The ridge regression was used to train the prediction model on the GDSC cell line dataset and the prediction accuracy was calculated via 10-fold cross-validation. According to the prediction model, the half-maximal inhibitory concentration (IC_50_) value for each ccRCC sample in the TCGA dataset was calculated. It was found that subtypes C2 and C3 are more sensitive to sorafenib and sunitinib drugs compared to subtype C1, meaning that subtypes C2 and C3 benefit from chemo drug treatment (Figure 9). Moreover, we probed the likelihood of immunotherapy response by subtypes, and the result revealed that subtype C2 (90/259 = 0.347) and C3 (25/38 = 0.658) are likely to respond to immunotherapy compared to C1 (34/215 = 0.149) (Fisher's Exact Test P-value = 1.424e-11). Notably, due to the small sample size of subtype C3, the accuracy of these findings needs to be further validated.

## Discussion

Previous studies are mainly based on the immune genomic profiling to reveal ccRCC molecular subtypes, and no studies using the tumor suppressor gene expression to explore the classification of ccRCC 21. Therefore, to fill this knowledge gap, we narrowed on identifying the potential molecular subtypes of the tumor suppressor gene based on the genomic expression profile. Our findings demonstrated that ccRCC can be categorized into three stable molecular subtypes based on the TSGs i.e., C1, C2 and C3. Moreover, using WGCNA analysis, a total of six significant modules were identified. Results from GO and KEGG enrichment analysis showed that the green module which positively associated with C1 and negatively associated with C2 and C3, was enriched in immune-related pathways, such as B cell receptor signaling pathway, Th1, and Th2 cell differentiation and Th17 cell differentiation. In addition, cancer-related (the p53 signaling), cell cycle, Hippo signaling, FoxO signaling and Wnt signaling pathways were enriched in other modules. These results suggesting that the genes of modules associated with distinct subtypes influence the development of ccRCC.

The tumor microenvironment (TME), which consists of malignant tumor cells, a variety of infiltrating immune cells, fibroblasts, and numerous chemokines, is a complex biological process 22. In the TME, immune response regulates tumor growth, invasion, and metastasis, hence, it is an alternative therapeutic target other than radiation and chemotherapy 23, 24. Our previous findings demonstrated that the immune-related pathways were enriched in the subtypes. Therefore, we further investigate the relationship between subtypes and immune infiltration levels. Surprisingly, subtype C1 was found to exhibit an enhanced immunity whereas subtype C3 harbored limited immunity in the TME of ccRCC. Moreover, the correlation between subtype and immune/stromal score were analyzed and discovered an increasing trend from C3 to C1 in the immune score and stromal score. Importantly, the subtype survival result showed that C3 and C1 corresponded to a poor survival outcome, while C2 corresponded to a better survival outcome implying that high levels of infiltrating immune cells contribute to the poor outcomes in ccRCC.

Conventionally, depending on the stage and site, treatment of ccRCC involved surgery, radiation, and chemotherapy. Whilst acknowledging the steady enhancement of treatment methods, the curative effect is limited, especially in recurrent and metastasis of ccRCC. This calls for further research to unearth the potentiality of target compounds in ccRCC treatment. To identify potential compounds for the treatment of ccRCC, 99 DETSGs (55 up-regulated and 44 down-regulated) were uploaded to the CMAP database, and 59 compounds were obtained. Among the 59 compounds, a few have been confirmed to be associated with the treatment of RCC. The leflunomide (LEF) is an inhibitor of dihydroorotate dehydrogenase (DHODH) that broadly used in the prevention and treatment of autoimmune disorders and allograft rejection, also approved for the treatment of rheumatoid arthritis 25. For instance, a study by Chen *et al* reported that LEF significantly minimizes cell proliferation of renal carcinoma cells in a manner dependent on concentration 26. Moreover, LEF treatment significantly inhibits the FZD10 (receptor mediating WNT/β-catenin activation) expression level and elsewhere, vivo xenograft experiment demonstrated inhibitory effects of LEF on tumor growth and Wnt/β-catenin signaling 26. Naftopidil is an adrenergic receptor antagonist which possesses growth inhibitory effects on human prostate cancer cells 27. A study by Iwamoto *et al* discovered that naftopidil promotes G1 cell-cycle arrest and decreases microvessel density in renal cell carcinoma, which might act as a novel anticancer agent for RCC 28. Ribavirin is an antiviral that has been reported to hinder proliferation, migration, and induced apoptosis in RCC by inhibiting translation and activity of eIF4E-regulated protein 29. Despite the potential use and pharmacological features of the three compounds being validated, the chemotherapy impacts to ccRCC patients need to be investigated through clinical trials.

The clinical application of chemotherapy drugs including Sorafenib and Sunitinib that is broadly applied in the treatment of metastatic RCC patients. In that respect, we further estimated the sensitivity of the two drugs using the GDSC database, and imputed that subtype C2 is more sensitive to these drugs compared to subtype C1. Furthermore, we probed the probability of immunotherapy response by the three subtypes. Delightfully, we discovered that subtype C2 is likely to respond to immunotherapy than subtype C1. Therefore, these findings might partially give reasons why subtype C2 exhibits a better prognosis in ccRCC.

Generally, we uncovered 3 robust subtypes of ccRCC based on the expression of TSGs, which might offer guidance to clinicians in providing customized treatment and better understand the prognostic difference of various subtypes. However, several limitations are worth noting. First, the sample size of each subtype was small in the external validation dataset, resulting in an insignificant survival result or high false rate. Therefore, a larger sample size dataset needs to validate the reliability in future studies. Secondly, more experimental evidence is necessary to boost the reliability and stability of the subtypes, pathways, and expression of the TSG genes. Additionally, the sample size of subtype C3 was relatively low, which might invalidate the results of drug sensitivity and immunotherapy. Hence, we need to expand our sample size to verify these findings.

In conclusion, we discovered and verified three subtypes of ccRCC hinged on expression profiles of TSGs. These subtypes were associated with distinct immune infiltration levels and the outcomes of patients. As we know, this is an inaugural study to systemically elaborate the relationship between molecular subtypes of TSG and ccRCC. Besides, 59 potential compounds were predicted using the CMAP database whereby leflunomide, naftopidil, and ribavirin were the most prospective compounds for the treatment of ccRCC. We also discovered subtype C2 was more sensitive to sorafenib and sunitinib than other subtypes. These findings will provide critical guidance to clinicians regarding the prognosis of different molecular subtypes for developing novel strategies for the treatment of ccRCC.

## Supplementary Material

Supplementary figures and tables.

## Figures and Tables

**Table 1:** Clinical statistics of the 512 ccRCC patients among the three subtypes in TCGA dataset. The p value was calculated by performing chi-square test or fisher test and further corrected by Bonferroni test

Clinical parameters	Molecular subtypes (n, %)			*p* value	FDR
	C1	C2	C3		
**Age**					
<60	99	121	22	0.3902838	0.4878548
≥60	116	138	16		
**T stage**					
T1-2	119	180	29	0.0015863	0.0044308
	T3-4	96	79	9	
**Race**					
White	194	234	25	0.0002154	0.0010770
Asian	4	2	2		
Black or African American	17	23	11		
**Stage**					
Stage I	87	147	22	0.0017723	0.0044308
Stage II	24	26	6		
Stage III	62	54	2		
Stage IV	42	32	8		
**Radiation**					
Yes	2	3	0	0.7913667	0.7913667
NO	213	256	38		
**Pharmaceutical**					
Yes	32	34	7	0.644558	0.7161756
NO	183	225	31		
**Grade**					
G1	2	8	1	< 0.0001	< 0.0001
G2	77	129	13		
G3	81	108	14		
G4	54	14	5		
GX	1	0	5		
**Smoking**					
1-year	106	131	28	0.1266751	0.2111252
2-year	13	9	3		
3-year	80	97	6		
4-year	11	14	1		
5-year	5	8	0		
**Gender**					
FEMALE	51	112	13	4.90E-05	0.0004796
MALE	164	147	25		
**N**					
N0	91	115	22	0.03698	0.0739600
N1	11	3	2		
NX	113	141	14		
**M**					
M0	163	215	28	0.1552	0.2217143
M1	41	30	7		
MX	11	14	3		
