# Supplementary material for: Classification of Clear Cell Renal Cell Carcinoma based on Tumor Suppressor Genomic Profiling
Source: J Cancer. 2021 Feb 22;12(8):2359–70. doi: 10.7150/jca.50462 (PMC7974878; doi:10.7150/jca.50462)
Supplement: Supplementary file 1 — Supplementary figures and tables. [file jcav12p2359s1.pdf]

Supplementary materials

Supplementary Figure

Figure S1

Validation of tumor suppressor gene-related subtypes of ccRCC in ICGC dataset. (A) The cumulative distribution function (CDF) curves, which can described the probability distribution of a real random variable, and established using consensus clustering approach. CDF curves of consensus scores was calculated according to the different subtype number (k = 2, 3, 4, 5, 6, 7, 8, 9). (B) The CDF Delta area curve of ccRCC samples when k=3. (C) The heatmap of consensus matrix for three subtypes obtained by estimating of CDF curves. (D) Principal component analysis (PCA) of gene expression profile of the top 100 variance genes. Each sample is represented with a single point, with different color for each of the three subtypes.

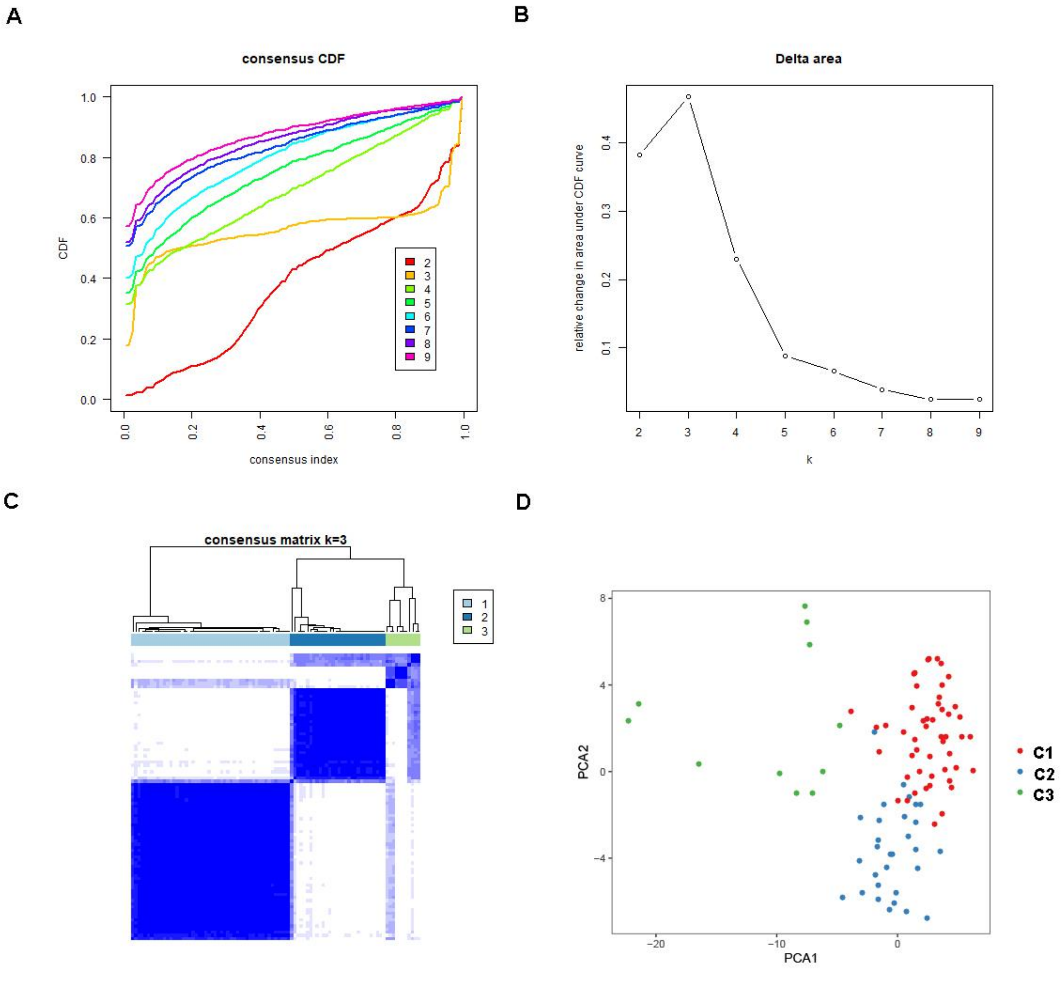

Figure S2

Heatmap and clinical features of the three subtypes in ICGC validation dataset.

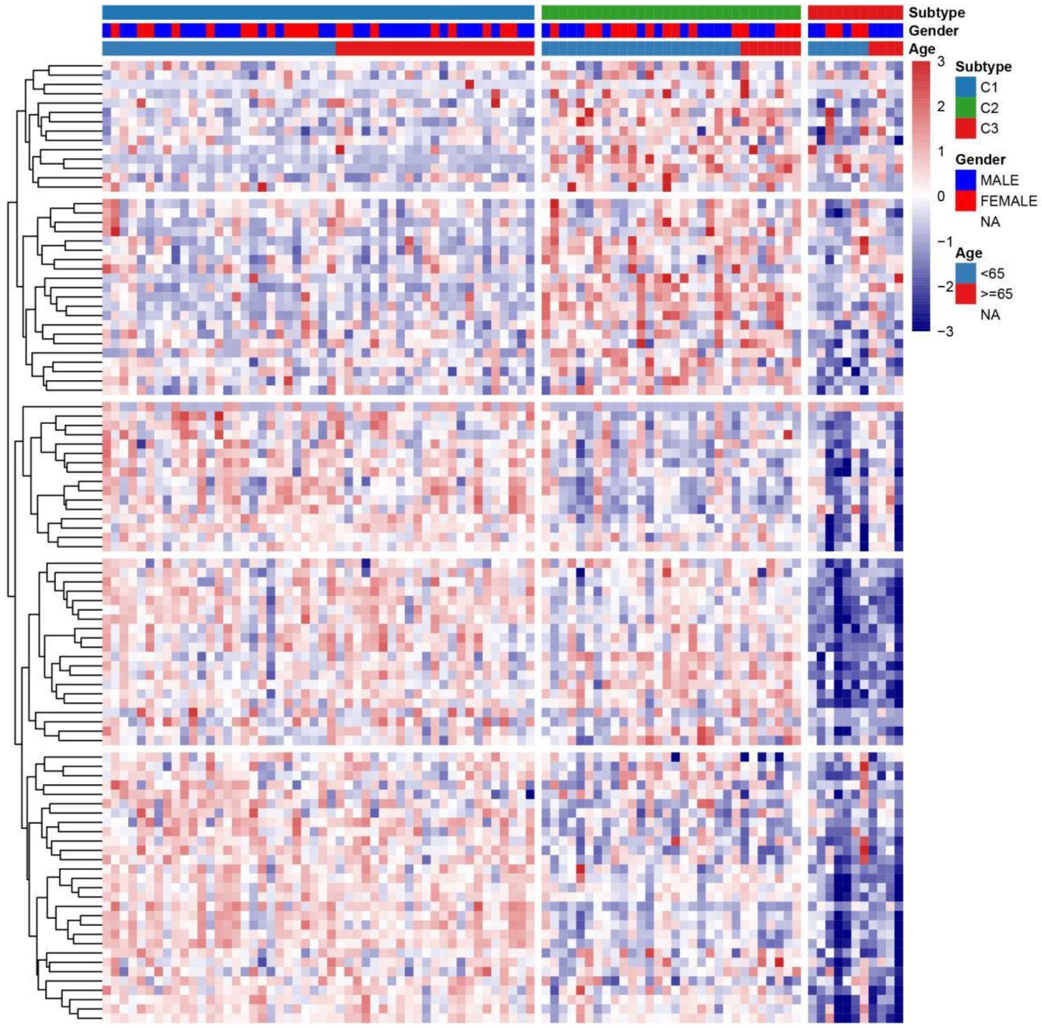

Figure S3

The SNP alteration of the three molecular subtypes

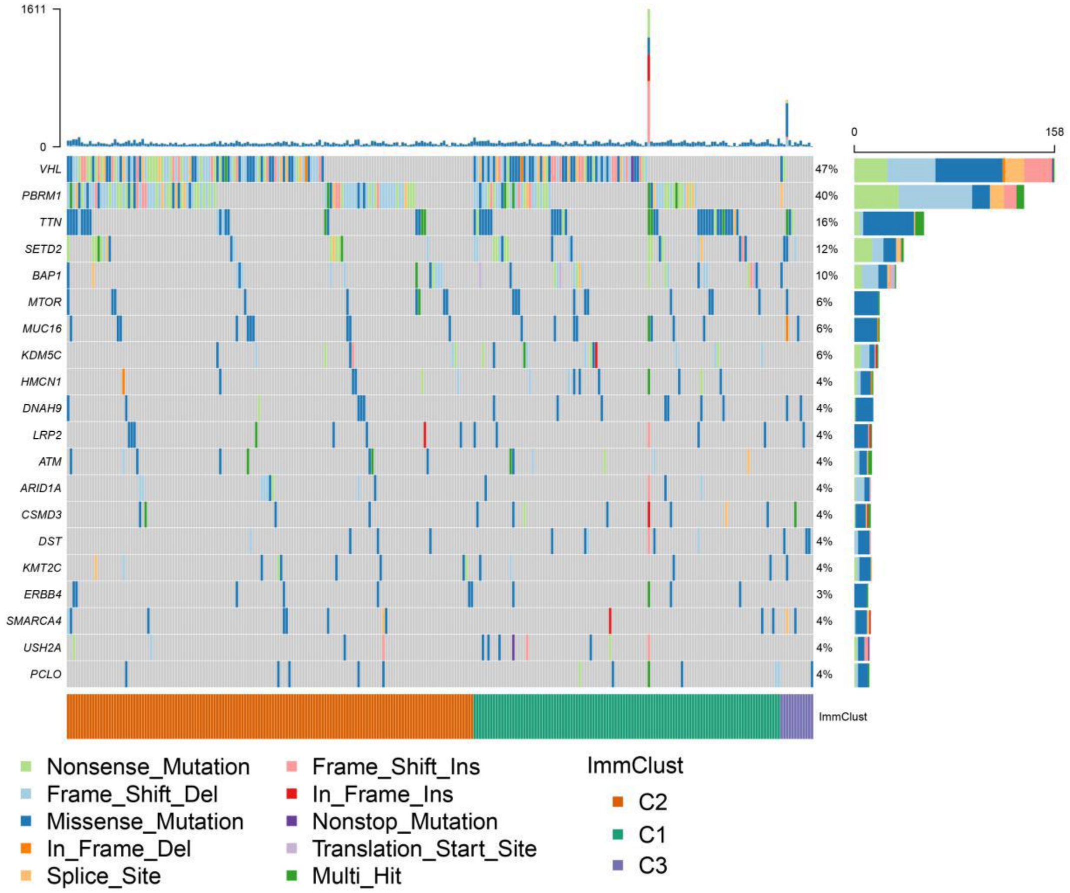

Figure S4

Gene Set Variation Analysis (GSVA) between the subtype C2 and subtype C1-C3 using the c2.cp.v7.1.symbols.gmt file as reference dataset

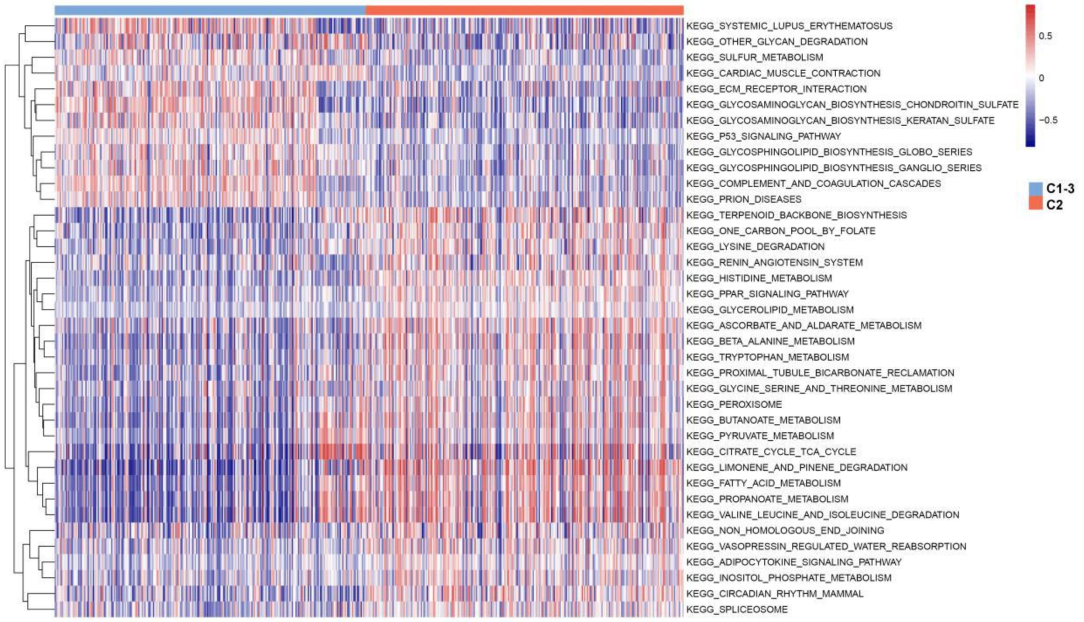

Figure S5

Clustering of samples and removal of outliers

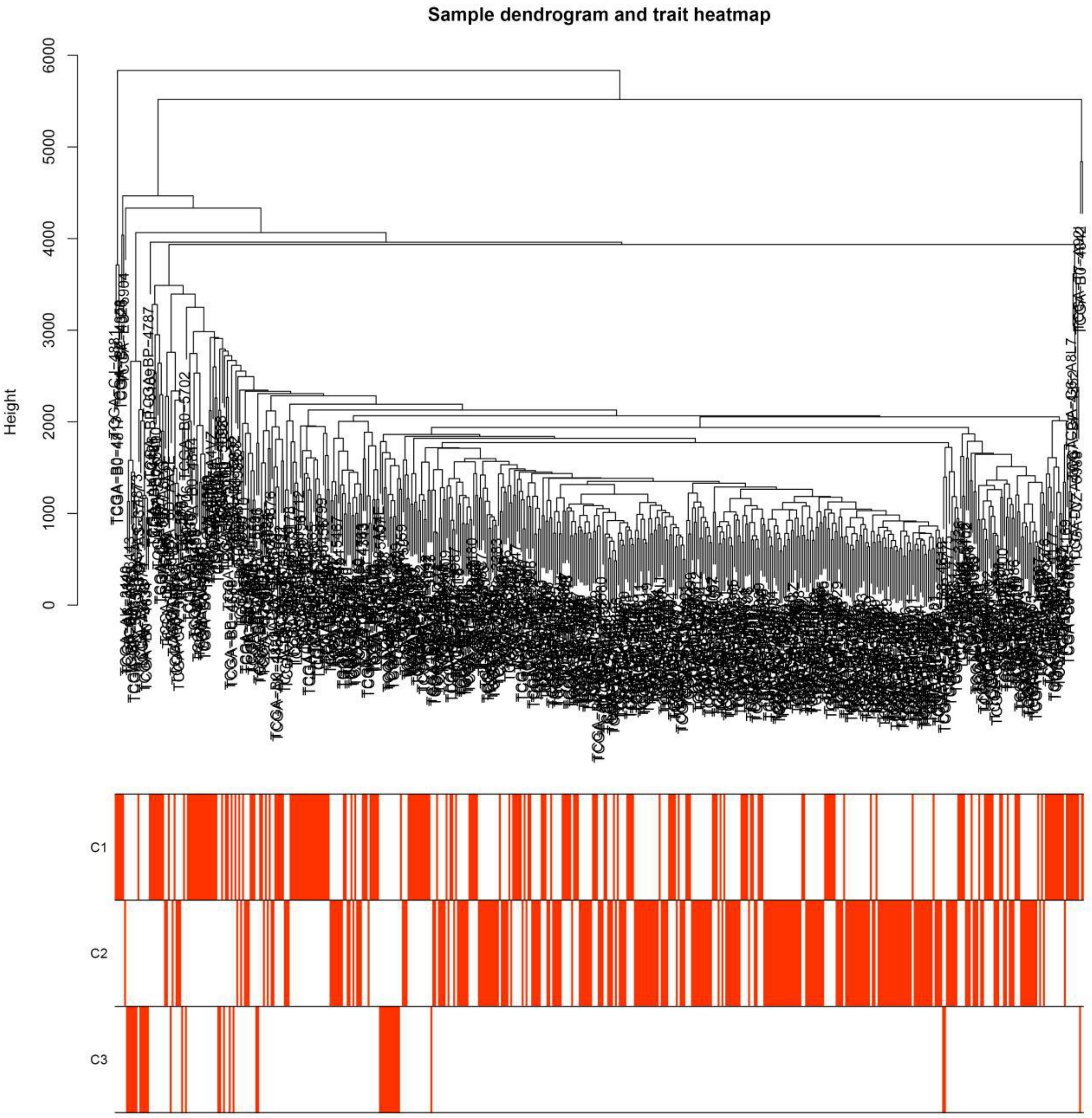

Figure S6

Cytoscape representation of the enriched pathways associated with co-expressed genes in the six modules.

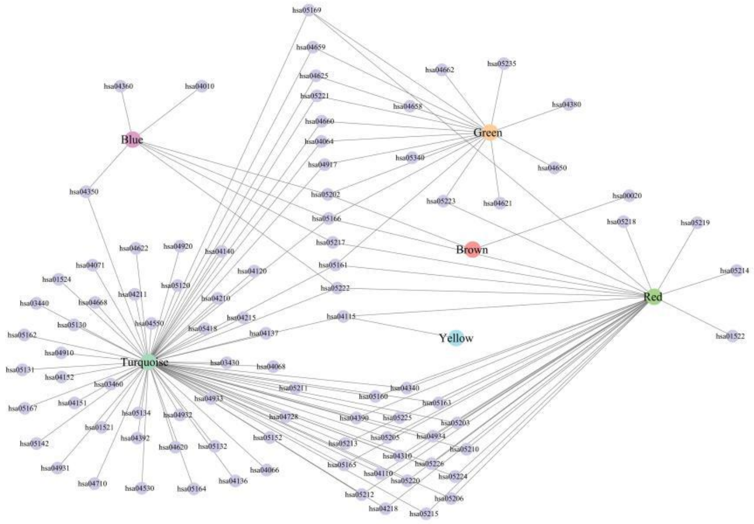

Figure S7

The overall expression level of the immune check point among the three subtypes

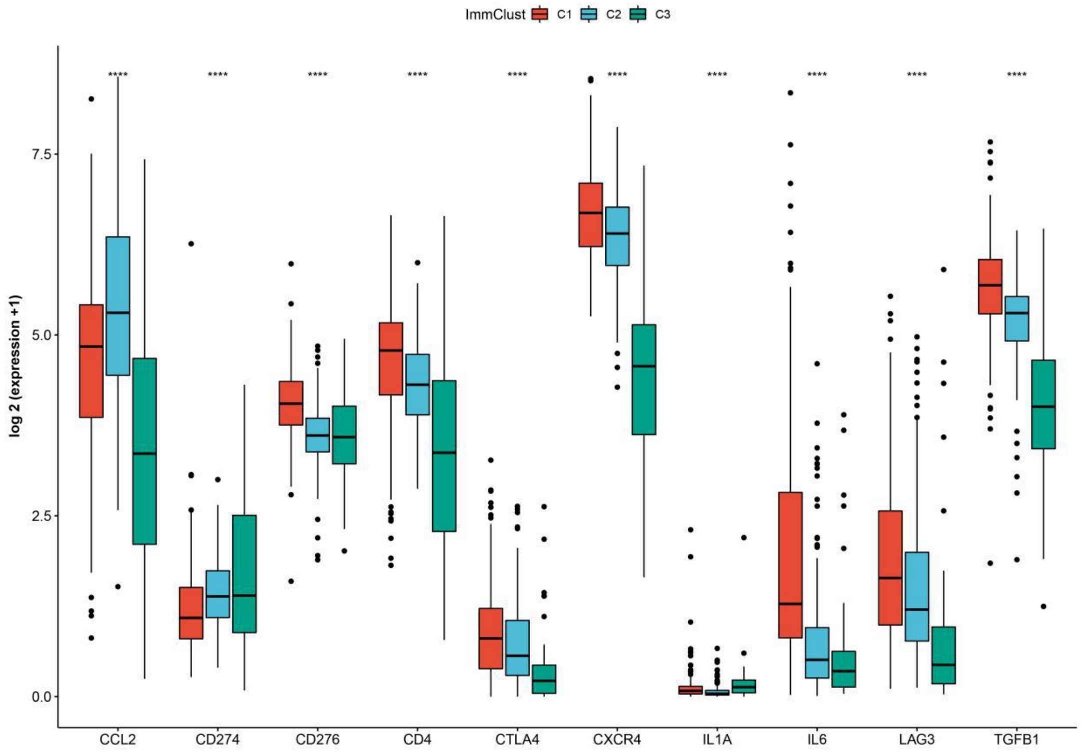

Figure S8

Heatmap for the up-regulated genes (A) and down-regulated genes (B) between

subtype C2 and subtype C1-C3

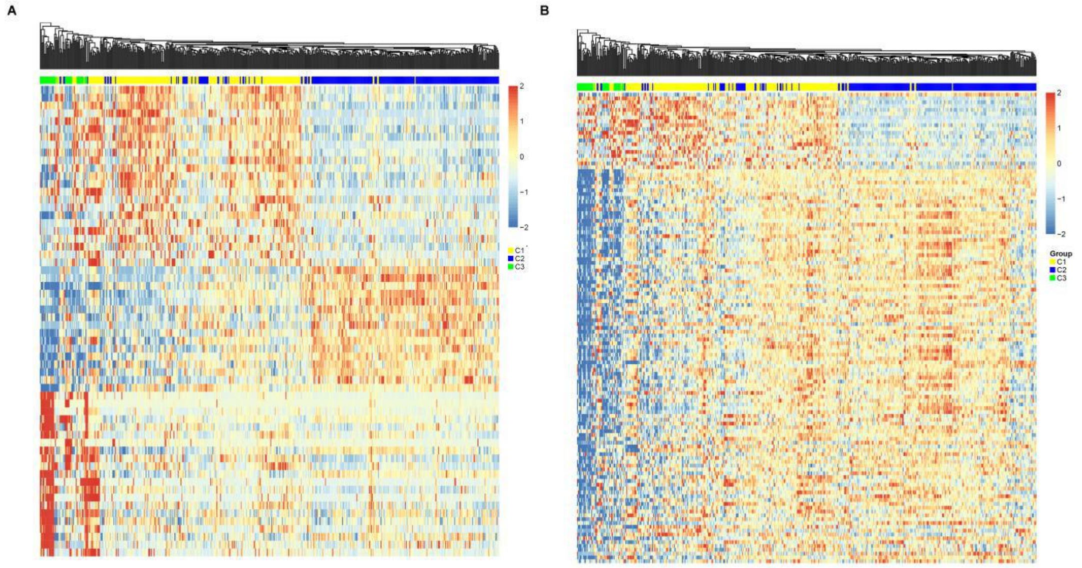

Supplementary Table

Table S1

compounds information that identified by using CMAP database based on differentially expressed TSGs.

cmap name	mean	enrichment	p	specificity
salsolinol	-0.518	-0.873	0.00409	0
pindolol	-0.411	-0.845	0.00026	0
penbutolol	-0.343	-0.832	0.00953	0.0074
mimosine	-0.306	-0.831	0.00973	0
PHA-00846566E	-0.351	-0.829	0.01	0
felbinac	-0.282	-0.814	0.00215	0.0426
nystatin	-0.428	-0.804	0.01526	0.0216
melatonin	-0.244	-0.757	0.00712	0.0256
aconitine	-0.295	-0.757	0.00716	0.0126
cloxacillin	-0.266	-0.747	0.00818	0.0242
metampicillin	-0.299	-0.737	0.00256	0.0073
mefexamide	-0.412	-0.729	0.01104	0.0067

propranolol	-0.2	-0.69	0.01995	0.0222
harpagoside	-0.247	-0.676	0.02449	0.0637
piperacetazine	-0.278	-0.669	0.02686	0.0292
nadide	-0.297	-0.656	0.03223	0.0523
raubasine	-0.419	-0.648	0.0366	0
tocainide	-0.24	-0.645	0.03776	0.1355
nizatidine	-0.389	-0.644	0.03831	0.0791
podophyllotoxin	-0.263	-0.638	0.04144	0.2843
CP-944629	-0.398	-0.637	0.04176	0.0879
suramin sodium	-0.4	-0.63	0.04597	0.0181
metixene	-0.167	-0.628	0.04691	0.0923
dehydrocholic acid	-0.313	-0.598	0.03016	0.0217
PNU-0251126	-0.256	-0.547	0.03371	0.1067
etiocholanolone	-0.137	-0.543	0.03621	0.2792
terguride	0.394	0.525	0.01388	0
urapidil	0.272	0.625	0.04963	0.0211
ribavirin	0.46	0.63	0.04645	0.1063
retrorsine	0.267	0.631	0.04625	0
gabapentin	0.541	0.632	0.04559	0.0076
leflunomide	0.456	0.632	0.04573	0.0848
sulfinpyrazone	0.325	0.638	0.04193	0.0256
(-)-MK-801	0.323	0.639	0.04132	0.0236
amrinone	0.488	0.641	0.04064	0.0441
pyrazinamide	0.309	0.667	0.02737	0.0315
glibenclamide	0.278	0.67	0.02642	0.0354
sulfametoxydiazine	0.284	0.676	0.02433	0.0472
meropenem	0.306	0.683	0.02166	0.0081
bisacodyl	0.348	0.691	0.01886	0.0851

ketorolac	0.499	0.703	0.01611	0
ricinine	0.346	0.705	0.01562	0.0069
etynodiol	0.244	0.705	0.01574	0.0373
pempidine	0.32	0.708	0.00509	0.0067
etamsylate	0.314	0.709	0.01472	0.0273
flavoxate	0.422	0.72	0.01241	0
quinisocaine	0.326	0.723	0.01182	0.0214
guanethidine	0.515	0.738	0.03515	0.024
sulfaphenazole	0.36	0.762	0.00605	0
naftopidil	0.451	0.801	0.01626	0
pivampicillin	0.472	0.819	0.00187	0
abamectin	0.356	0.823	0.00161	0
zomepirac	0.297	0.823	0.00165	0.0098
harmine	0.354	0.828	0.00133	0
biotin	0.565	0.836	0.00889	0
blebbistatin	0.629	0.845	0.04815	0.122
5279552	0.495	0.849	0.04585	0.0486
puromycin	0.625	0.876	0.00034	0.0955
benzbromarone	0.624	0.888	0.00276	0
